# Supplementary material for: Learning curve and surgical outcome of robotic assisted colorectal surgery with ERAS program
Source: Sci Rep. 2022 Nov 29;12:20566. doi: 10.1038/s41598-022-24665-w (PMC9709162; doi:10.1038/s41598-022-24665-w)
Supplement: Supplementary file 1 — Supplementary Information 1. [file 41598_2022_24665_MOESM1_ESM.zip › LSC Raw data-Ñ[▒K/075/H_ñw╝╨░O▒Kñσ.pdf]

正式摘要(電子簽章：000000189673045)	【讀取】憑證
W52 / W52 英文標題(注意:無英文姓名-請補病人基本檔!)	

臺中榮民總醫院

出院病歷摘要

轉入號： 轉出號：

[redacted]

(8)流水編號	
(9)入院日期	109/03/24 (2020) 直腸外科 W52 - 162
(10)轉科日期	
(11)出院日期	109/04/25 (2020) 住院天數計 32 日

診斷

- 入院:1.Rectal adenocarcinoma
  2.Hypertension
- 出院:1.Rectal adenocarcinoma, pT2N2b, s/p LAR+coloanal anastomosis+loop ileostomy on 109/03/26
  2.AfRVR, suspect hypovolemic status
  3.Hypertension
  4.Cholecystitis s/p PTGBD on 2020/4/21

主訴

- bloody stool and difficulty stool passage since 3 weeks ago

病史

- This 91 years old female has history of 1.hypertension 2. s/p hysterectomy
  She complained of bloody stool and difficulty stool passage since 3 weeks ago, then she went to LMD to receive colonoscopy, which revealed rectal cancer, and pathology proved adenocarcinoma. This time, she was admitted for preop survey and operation

體檢發現

- Anal inspection:
  Recto digital examination: ANTERIOR WALL, 5CM ABOVE ANAL VERGE
  Sigmoidoscopy:
  Anoscopy:
  Abdomen:
  - General appearance :
    - easy looking , conscious clear , without cardiopulmonary distress
    - Vital signs自動帶入date 體溫(BT) 脈搏(P) 呼吸(R) 血壓(BP) 身高(BH) 體重(BW) BMI
      20200324 36.6℃ 82次/分 17次/分 162/73mmHg 143.6cm 55.2kg 26.8
    - Vital signs: B.P. 162/73 mmHg , P.R. 82 /min , R.R. 17 /min , B.T. 36.6 ℃
  - HEENT :
    - Eyes: no anemic conjunctiva, no icteric sclera, normal EOM
    - ENT: no otorrhea, no rhinorrhea, no erythematous mucosa, no injected tonsil, no deformity
  - Neck: supple, freely movable, trachea midline,no deformity, no engorged jugular vein , no palpable thyroid or lymph node enlargement
  - Chest:
    - Inspection: Grossly normal, symmetric and fully expansion, no accessory muscle used, no operation scar, no spider angiomata, no gynecomastia
    - Palpation: no palpable mass or nodule, no subcutaneous emphysema, no tenderness
    - Ausculation: clear breathing sound
  - Heart:
    - Palpation: no thrill, no heave
    - Percussion: normal heart size
    - Ausculation: regular heart beat, normal S1, S2, no gallop, no murmur
  - Extremities: No deformity, full ranged of motion without limitation, no clubbing finger or nail cyanosis, no pitting edema, no ulcer or pigmentation, no obvious skin lesion, no varicose vein, no bone, joint or muscle tenderness

  CRS CONDITION
  - Karnofsky performance status: (100) Normal, no complaints, no evidence of disease
  - PE of Abdomen:
  - __1. Inspection: LOW ABDOMINAL SCAR DUE TO PREVIOUS HYSTERECTOMY
  - __2. Ausculation: NORMAL ACTIVEBS
  - __3. Palapation: NO PALPABLE TUMOR
  - __4. Percussion: NO OBVIOUS TYMPACNIC SOUND
  - CEA: 4.27 ng/ml
  - Imaging Studies:
  - Colonoscope report: rectal cancer
  - Figures:
    1. colon & rectum

手術日期及方法

- 手術日期及方法20200326 Low anterior resection with colo-anal anastomosis+ Ileostomy+ Laparoscopy

住院治療經過

- During admission, LAR with coloanal anastomosis and loop ileostomy were arranged on 109/03/26.
  After operation, due to old age, she was transfered to SICU for further care. In SICU, we kept post-operative care with pain control, tube drainage, and wound care.
  Endotracheal tube was remove on 3/27. And she resumed water intake at same day.
  NG tube was removed on 3/28, however, she complained abdominal fullness with nausea sensation.
  Hence we replaced NG tube with decompression.
  Sometimes AfRVR without chest pain was noted, suspect hypovolemic related, we used fluid supplement and Bisoprolol, Amiodarone prn use and the symptom relieved gradually.
  Under her relative stable condition, she was transferred to ordinary ward on 03/31. In ward, she resumed oral intake since 4/1 without post-prandial discomfort initially.
  However, abdominal fullness was noted on 4/4, and KUB showed ileus favor displaced penrose drain related.
  Hence drain was removed at same day. Bue follow KUB still showed ileus. We consult PPN and prescribed smofkabiven.
  Her condition improved gradually with softer abdomen, fair activity, and more stool passage. CT on 4/13 revealed no obvious abnormality but dilated GB.
  There was no murphy signe nor fever.
  But PE still revealed dilated abdomen without pain or tenderness. Sono on 4/20 showed dilated GB.
  After discussed with patient, PTGBD was arranged on 4/21. She mentioned soft abdomen with less distension gradually.
  Base on her stable condition, she wasa rranged discharge with OPD follow up.

合併症

一般檢查

- 累積報告：

DATE	TIME	ALB	TP	BIL.T	BIL.D	ALKP	AST	ALT	LDH	CHOL	TG	HDL-C	LDL	UA	NH3	AMY
1090325	0922	4.0		0.3				36								
1090326	1948			0.3	0.1											
1090327	0530	3.1		0.5	0.1		30	25								
1090329	0532			0.3	0.1											
1090330	1120									133	56					
1090331	0506			0.25				11								
1090402	0709	2.8		0.15				16								
1090406	1141									151	1352					
1090409	0923	3.0		0.28				22			194					
1090413	0923	3.0		0.43	0.15		17	11								
1090416	0907	3.3		0.26				11								
1090420	0924			0.44	0.12		28	13								
1090422	1330	3.4		0.51	0.16		23	13								

DATE	TIME	WBC	RBC	HGB	HCT	MCV	PLT	BLST	NEUT	BAND	SEG	LYM	MON	EOS	BAS
1090325	0857	3990		12.6			220		55.9			31.8	6.0	5.0	1.3
1090326	1946	9840	4.14	11.3	35.5	85.7	215		89.9			7.3	2.3	0.2	0.3
1090327	0528	11260		10.5			208		93.8			4.7	1.3	0.0	0.2
1090328	1437			10.1	32.6										
1090329	0531	7690	3.66	9.9	32.0	87.4	189		73.4			18.7	5.1	2.1	0.7
1090329	1813			11.6	36.6										
1090331	0505	7580		11.4	36.3		225		69.9			19.9	5.1	4.2	0.9
1090402	0703	6650	4.05	11.1			200		69.9			19.1	6.3	4.1	0.6
1090404	1843	8810	4.57	12.3	38.3	83.8	257		84.2			8.1	5.8	1.6	0.3
1090409	0856	8290	4.06	11.2			348		80.0			10.7	4.9	3.9	0.5
1090413	0923	6680	4.07	11.4	34.1	83.8	374		77.8			13.0	5.2	3.7	0.3
1090416	0909	7260	4.16	11.4			388		80.0			12.1	5.4	2.1	0.4
1090420	0935	5010	4.38	11.9	37.3	85.2	329		67.4			23.0	5.4	3.4	0.8
1090422	1330	10650	3.94	10.7			275		89.1			7.6	3.1	0.0	0.2

DATE	TIME	NA	K	CL	CA	BUN	CREAT	Mg	P	CRP	ESR	PCT	LIPASE	LACTATE
1090325	0922	140	4.7			10	0.65							
1090326	1948	139	3.2				0.65							
1090327	0530	141	3.6			14	0.79							
1090328	1438	141	3.4		7.7			1.9	1.6					
1090329	0532	140	3.3				0.56	1.8	2.2					
1090329	1815	135	3.8		8.1									
1090331	0506	139	3.9	105	8.5	10	0.59							
1090331	0652													6.5
1090402	0709	136	4.5	104	7.9	11	0.62							
1090404	1842	134	4.3	100	8.4	14	0.55	2.1		2.085				12.3
1090406	1141							2.7	6.1					
1090409	0923	134	4.6	98	8.2	12	0.58							
1090413	0923	131	4.3		8.2	12	0.53							
1090413	1620							2.1	3.3					
1090416	0907	131	4.5	93	8.7	20	0.61							
1090420	0716													5.8
1090420	0924	135	4.4		8.8	10	0.62							
1090422	1330	133	3.9	100	8.3	10	0.66							

DATE	TIME	GLUCOSE	PAB	TRF	ZN	CU	KETONE	CK	CKMB	PT-P	PT-C	APTT-P	APTT-C	TRO-T
1090328	1438							31	4					
1090328	1439													33.08
1090329	1815								4					
1090329	1819													30.59
1090420	1642									10.8	10.3	24.8		

DATE	TIME	SP-GR	PH	PROT	GLUC	KETON	BILI	UROB	OB	NITR	LEU	RBC	WBC	EPCEL
1090416	2022	1.016	8.0	2+	Negative	Negative	Negative	0.2	3+	Positive	2+	+	10-20	2-4
1090422	1448	1.017	6.5	2+	Negative	Trace	Negative	0.2	1+	Positive	3+	5-10	3+	6-10

DATE	TIME	PH	PCO2	PO2	SO2	BEB	HCO3	TCO2	HGB	FIO2
1090326	1946	7.337	42.3	347.4	99.9	-3.4	22.2	23.5		
1090329	0535	7.402	38.7	123.6	98.7	-1.0	23.6	24.8		

DATE	TIME	PH	BEB	SCVO2	PO2	HCO3	HGB	PCO2	TCO2
1090404	1840	7.417	2.6	80.6	43.7	27.5		43.7	28.9

- 放射線報告：**檢驗項目 :Percutaneous gall bladder drainage 報告日期 :1090421**

主　訴：
Result:
Finding:
The procedure is done by R3 and covered under CR.
1. After informed consent was obtained, the patient was on supine position.
2. The GB and biliary system were examined by the sonography.
3. After the puncture point was selected, the depth, angle and level of the GB were determined.
4. The skin was prepared and draped in the usual sterile fashion.
5. 5 ml of 2% lidocaine was injected for local anesthesia.
6. A 18G PCN needle was used to puncture the gallbladder. The needle was removed and bile flowed out. Contrast media was injected to show the gallbladder.
7. A 0.038In. guidewire was inserted along the needle sheath and into the gallbladder.
8. Finally, a 10Fr. PTCCD pigtail tube was inserted along the guidewire into the gallbladder.
9. The PTCCD tube was secured and the wound was dressed. Image was taken for documentation.

Findings:
Some dark greenish bile was aspirated out from gallbladder.
Impression:
The PTCCD had been performed as described above.

檢驗項目 :CT ABDOMEN-SURVEY 報告日期 :1090415

主　訴：
Result:
Finding:
CT scan of abdomen with administration of IV and oral contrast agents:
> History of rectal cancer S/P LAR, coloanal anastomosis and loop ileostomy
> Dilatation of small intestine is noted. Suggest check function of the ileostomy.
> Small low-attenuation lesions in S4 of liver, hepatic cysts are suspected.
> Marked distention of gallbladder without wall thickening or surrounding fat stranding, suggest check Murphy sign.
> Bilateral renal cysts
> A fat-content mass about 4.3cm is noted in left adrenal gland, in favor of myelolipoma.
> Mild ascites in pelvis
Impression:

- 1. Small bowel obstruction, suggest check function of the ileostomy.
  2. Marked distention of GB, suggest check Murphy sign.

特殊檢查

病理報告

- 活體組織切片病理報告　手術日期：**109/03/26**　病理號：**S0908968**

  診斷醫師:陳律臻 報告日期:109/04/08
  Pathologic diagnosis:
  1. Rectum, lapa low anterior resection --- Adenocarcinoma, moderately to poorly differentiated. Tumor invaded to muscularis propria and metastasized to perirectal lymph nodes (17/26). The bilateral cut ends are free of tumor.
  2. IMA root, dissection --- Unremarkable fibrovascular tissue.
  3. Rectum, cut end 1, distal, resection --- a. Chronic inflammation.
  b. Lymphovascular tumor thrombi in perirectal soft tissue.
  4. Rectum, cut end 2, distal, resection --- Chronic inflammation.
  Ancillary study for diagnosis:
  IHC stains for MLH1, MSH2, PMS2 and MSH6 are done on section C. All above markers are preserved.
  Prognostic and predictive factor:
  1. Tumor size: 2.4x1.9 cm.
  2. Depth of invasion: Tumor invades to muscularis propria.
  3. Histologic grade: G3: Poorly differentiated.
  4. Angiolymphatic invasion: Present, extensive.
  5. Perineural invasion: Not identified.
  6. Tumor deposits (discontinuous extramural extension): Not identified.
  7. Macroscopic tumor perforation: Not identified.
  8. Surgical Margins: a) Proximal Margin: Uninvolved by invasive carcinoma, distance of tumor from margin: 15 cm. b) Distal Margin: Uninvolved by invasive carcinoma, distance of tumor from margin: 1 cm. c) Radial or Mesenteric Margin: Uninvolved by invasive carcinoma, distance of tumor from margin: 1 mm.
  9. Lymph node status: Involved (18/27).
  10. Extranodal involvement: Present.
  11. Tumor regression grading S/P CCRT: No known presurgical therapy.
  12. Type of polyp in which invasive carcinoma arose: Not identified.
  13. Additional pathologic findings: Not identified.
  14. Pathological TNM Stage: pT2N2b(According to the eighth edition, American Joint Committee on Cancer Staging Guidelines for Tumors).
  15. TNM descriptors: Not applicable.
  Gross description:
  The specimen consists of 1) a segment of colon, 17 cm in length and 3 cm in proximal cut end circumference, 4.5 cm in distal circumference, and perirectal soft tissue. A gray tan firm polypoid tumor, 2.1x1.9 cm, is noted, 1 cm to the distal cut end. On cross section, the tumor invades to the muscle layer with 1 mm to the radial margin. The both cut ends are free of tumor grossly. 2) IMA root, a piece of tan yellow soft tissue, 3.2x2.5x1 cm. 3) distal cut end 1, a piece of tan mucosa-covered soft tissue, 2x1.6x0.6 cm. 4) distal cut end 2, a piece of tan mucosa-covered soft tissue, 1.8x1.6x0.5 cm. Representative part for section: A) proximal cut end B) distal cut end C-E) tumor F) colon G-J) specimen 2-4 K-N) lymph nodes.
  Note:
  1. The interpretation of MMR panel and recommended studies are as below: (1) No loss of nuclear expression of MMR proteins: low probability of microsatellite instability-high (MSI-H). (2) Please note: There are exceptions to the above IHC interpretations. These results should not be considered in isolation, and clinical correlation with genetic counseling is recommended to assess the need for germline testing.
  2. This case has been peer reviewed by two doctors.
  Erratum: 2020.04.24
  Prognostic and predictive factor:
  9. Lymph node status: Involved (17/26).
  #T-59600_2 #M-81403_2 3 1105 M0000F
  #T-C4560_2 #M-81406_2 3 1105 ER0000

其它

出院時情況

- 3.改門診治療

出院指示

藥名	劑量	劑量單位	用法	頻次	數量	數量單位	現況	天數	單位
Norvasc tab 5mg	1	TAB	PO	BID	6	TAB	TH	3	天
$Biofermin tab	2	TAB	PO	TID	18	TAB	TH	3	天
Anxiedin tab 0.5mg	0.5	TAB	PO	QNPRN	2	TAB	TH	3	天
Smecta powder 3g	1	PKG	PO	TID	9	PKG	TH	3	天
Gaslan tab 40mg	1	TAB	PO	QID	12	TAB	TH	3	天
MOSAD tab 5mg	1	TAB	PO	TID	9	TAB	TH	3	天
Megest susp 120mL	10	ML	PO	QD	1	ML	TH	3	天
Esomeprazole tab	1	TAB	PO	QDAC	3	TAB	TH	3	天
Vesicare tab 5mg	1	TAB	PO	QD	3	TAB	TH	3	天

出院計劃

- 門診日期
  - date on 2020/04/28

主治醫師_2062F 蔣錚帆_____ / 住院醫師_2327J 王郁棋_____
正式摘要(電子簽章：**000000189673045)**

W52 / W52

doctorRank:B
